# Defining a timeline of colon pathologies after keratin 8 loss: rapid crypt elongation and diarrhea are followed by epithelial erosion and cell exfoliation

**DOI:** 10.1152/ajpgi.00140.2023

**Published:** 2023-11-14

**Authors:** Maria A. Ilomäki, Lauri Polari, Carl-Gustaf A. Stenvall, Mina Tayyab, Kirah Kähärä, Karen M. Ridge, Diana M. Toivola

**Affiliations:** ^1^Cell Biology, Biosciences, Faculty of Science and Engineering, Åbo Akademi University, Turku, Finland; ^2^InFLAMES Research Flagship Center, Åbo Akademi University, Turku, Finland; ^3^Division of Pulmonary and Critical Care Medicine, Northwestern University, Chicago, Illinois, United States; ^4^Turku Center for Disease Modeling, University of Turku, Turku, Finland

**Keywords:** colon, exfoliated cells, keratin, mouse model, noninvasive

## Abstract

Keratins are epithelial intermediate filament proteins that play a crucial role in cellular stress protection, with K8 being the most abundant in the colon. The intestinal epithelial-specific K8-deficient mouse model (K8^flox/flox^;Villin-Cre) exhibits characteristics of inflammatory bowel disease, including diarrhea, crypt erosion, hyperproliferation, and decreased barrier function. Nevertheless, the order in which these events occur and whether they are a direct cause of K8 loss or a consequence of one event inducing another remains unexplored. Increased knowledge about early events in the disruption of colon epithelial integrity would help to understand the early pathology of inflammatory and functional colon disorders and develop preclinical models and diagnostics of colonic diseases. Here, we aimed to characterize the order of physiological events after *Krt8* loss by utilizing K8^flox/flox^;Villin-CreER^t2^ mice with tamoxifen-inducible *Krt8* deletion in intestinal epithelial cells, and assess stool analysis as a noninvasive method to monitor real-time gene expression changes following *Krt8* loss. K8 protein was significantly decreased within a day after induction, followed by its binding partners, K18 and K19 from *day 4* onward. The sequential colonic K8 downregulation in adult mice leads to immediate diarrhea and crypt elongation with activation of proliferation signaling, followed by crypt loss and increased neutrophil activity within 6–8 days, highlighting impaired water balance and crypt elongation as the earliest colonic changes upon *Krt8* loss. Furthermore, epithelial gene expression patterns were comparable between colon tissue and stool samples, demonstrating the feasibility of noninvasive monitoring of gut epithelia in preclinical research utilizing Cre-LoxP-based intestinal disease models.

**NEW & NOTEWORTHY** Understanding the order in which physiological and molecular events occur helps to recognize the onset of diseases and improve their preclinical models. We utilized Cre-Lox-based inducible keratin 8 deletion in mouse intestinal epithelium to characterize the earliest events after keratin 8 loss leading to colitis. These include diarrhea and crypt elongation, followed by erosion and neutrophil activity. Our results also support noninvasive methodology for monitoring colon diseases in preclinical models.

## Graphical abstract

**Figure:**
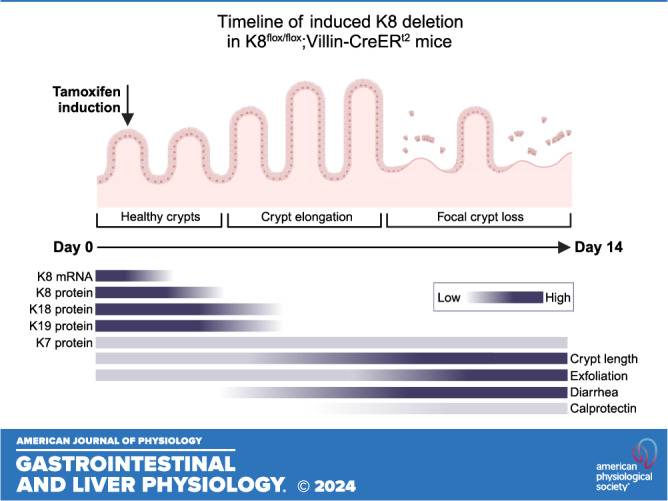

## INTRODUCTION

Cytoplasmic intermediate filament (IF) proteins form dynamic filaments through the assembly of tetramers equally constituting type I and type II keratin (K) units (1). These proteins maintain the mechanical properties of epithelial cells and link cell-cell and cell-basal lamina junctions via dense filament networks (2, 3). The keratin-cytoskeleton is essential for the integrity of enterocytes, which form a single-cell epithelial surface throughout the intestine (4). The common keratins expressed in a healthy mouse intestine are type I K18–20 and type II K7–K8, and the expression depends on the phase of cell differentiation. K8 is the most abundant type II keratin in the colon and small intestine, and K19 is the most abundant type I keratin (5, 6).

Loss of intestinal epithelial K8 in mouse colon leads to a downregulation of both K18 and K19 with no compensation by K7. The phenotype is characterized by diarrhea, hyperproliferation, increased sensitivity to induced tumorigenesis, epithelial erosion, compromised barrier integrity, and an inflammatory phenotype in aging mice (7). The intestinal specificity of K8 deletion is achieved using the Villin-Cre-1000 or Villin-CreER^t2^ system, with the villin promoter stably targeting the expression of the transgene in intestinal epithelial cells (8). It remains unclear whether the epithelial erosion observed in these mice is a mechanical consequence of either *1*) epithelial fragility and compromised ion transport, *2*) increased inflammatory activity due to a leaky barrier, or *3*) caused by an unknown mechanism as a response to the disruption of the cytoskeletal network (9). Phenotypes similar to K8 knockout mice have been reported in mice where other molecular components of the colonocyte cytoskeletal framework have been hampered. These include, e.g., conditional α6-integrin and plectin knockout mice, in the latter of which the cellular keratin distribution is altered, resulting in changes resembling those found in K8-deficient mice (10–12). The similarities between these knockout phenotypes might be related to mechanical fragility, a consequence of reduced connections between keratin filaments and other components of the cellular cytoskeleton and junctional complexes (13), for which the functional keratin network is required (14, 15).

The K8 knockout mouse has been referenced as a possible disease model for inflammatory bowel disease, IBD (16, 17). These mice have a few specific benefits compared with more commonly used IBD models as they are not immunocompromised and the manifestation of colitis is relatively modest (18). We recently found that the inflammatory activity was less pronounced in mice with colon epithelial cell-specific keratin deletion than the global K8 knockout mice, which manifest disease phenotypes in multiple organs (19, 20). This might indicate that the infection-inflammation axis is not a determinant for the keratin-deficient colon phenotype compared with other commonly utilized colitis murine models defined by elevated immune responses (18). K8-deficient mice survive up to ages over one year, thus reflecting more long-term chronic disease than acute inflammation (7, 19). The events that trigger IBD development in humans are not fully understood, and characterization of early events in conditional K8-loss could help fill this gap by modeling early disease development (21, 22).

Here, we utilized the recently described inducible and tissue-specific K8^flox/flox^;Villin-CreER^t2^ (7) mice that allow for the study of the sequential early events following K8 loss in adult mice after tamoxifen (TAM) administration, to understand the primary functions of K8 in the colonic epithelium. Daily molecular, cellular, and pathological changes as well as macroscopical colitis manifestation were investigated to study the sequence of events in the colon after conditional K8 deletion. In addition, we present a noninvasive stool sample-based method to determine gene expression changes and intestinal crypt loss in preclinical colon disease models.

## MATERIALS AND METHODS

### Study Setup and Experimental Animals

Mice were generated for Prof. Ridge (Northwestern University) by Ozgene (Cambridge, MA) as described by Stenvall et al. (7). Genotypes were determined using PuReTaq Ready-To-Go (RTG) PCR Beads (GE Healthcare, UK) and the primers 5′- GCGTGGCTTTGGGATTTAGATTAG-3′ and 5′- CCTCCAGCCATGTTTCTTTATCTC-3′ for the flox transgene and 5′- GCGATCGCTATTTTCCATGA-3′ and 5′- TCGATGCAACGAGTGATGAG-3′ for the Cre transgene. Mice were housed at the Central Animal Laboratory of the University of Turku with free access to a Teklad 2018 (Envigo) diet and water ad libitum, under a license (ESAVI/16359/2019 and ESAVI/4498/2023) issued by the State Provincial Office of South Finland, according to animal study protocol approved by the Finnish Animal Ethics Committee.

**Figure 1.**
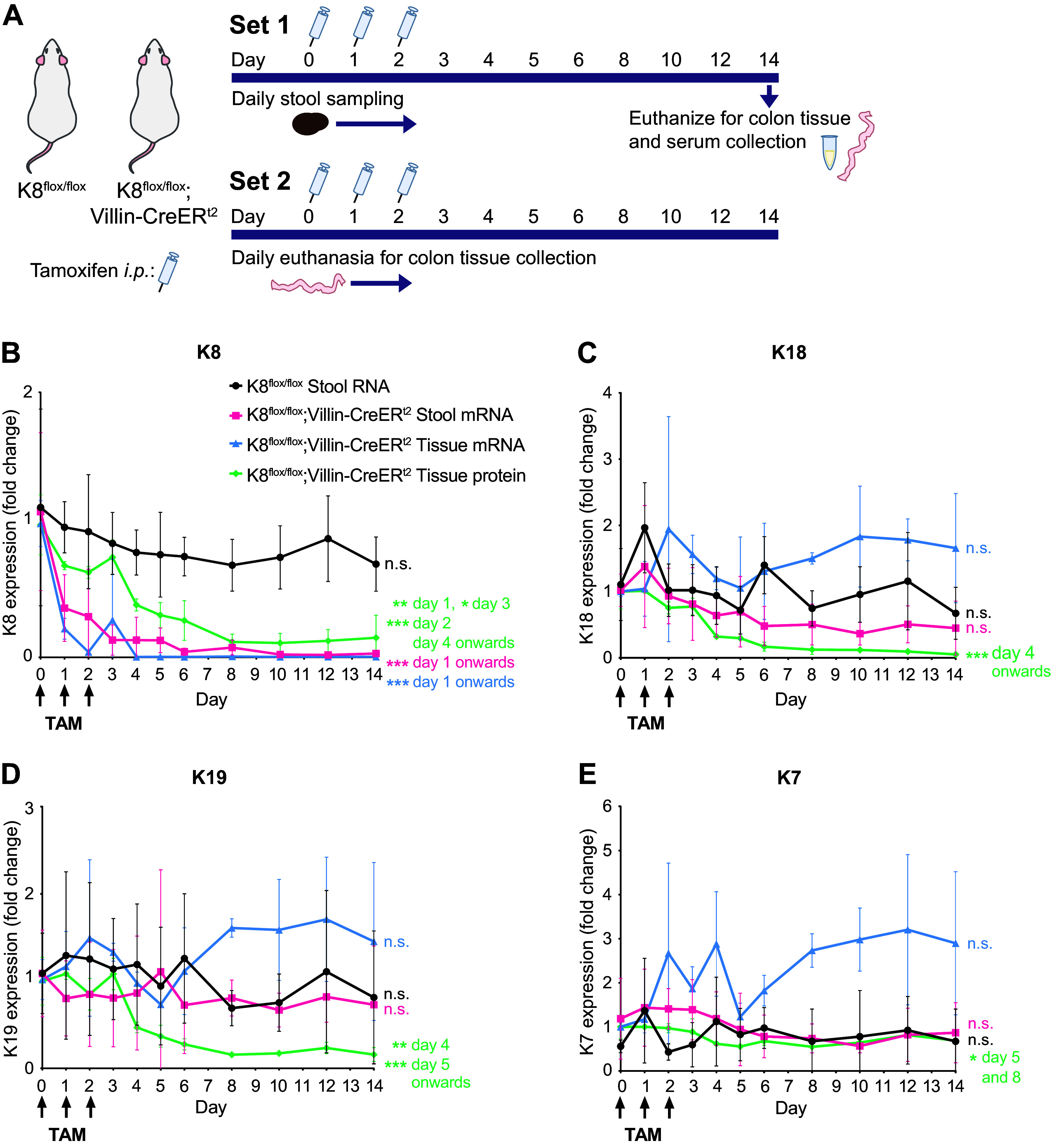
Targeted intestinal epithelial K8 deletion in mice leads to an immediate loss of K8 mRNA and protein, followed by a slower decline of K18 and K19 protein, as seen in both colon tissue and stool samples. *A:* schematic diagram of mouse experiments. K8 (*B*), K18 (*C*), K19 (*D*), and K7 (*E*) protein and mRNA expression levels were measured in lysates of crudely isolated colonic epithelium (scrapings) and stool samples from K8^flox/flox^;Villin-CreER^t2^ mice collected 0–6, 8, 10, 12, and 14 days following induction of colon-specific K8 loss with tamoxifen (TAM). K8^flox/flox^ stool samples were used as controls. Protein results were normalized to Hsc70 whereas mRNA expression was normalized to *Actb*. Tissue protein and mRNA levels were measured from mouse *set 2* (*n* = 3) with different mice/time point, whereas stool mRNA samples were collected from mouse *set 1* (*n* = 7) with the same mice in every time point. Dots indicate mean value with whiskers representing SD. Statistical significance comparing all the other days to *day 0* determined using two-way ANOVA Bonferroni’s post hoc test for *B–E*. Statistically significant differences are shown as *P < 0.05, **P < 0.01, and ***P < 0.001.

A 15 mg/mL tamoxifen (TAM) (Sigma-Aldrich, St. Louis, MO) solution was prepared by dissolving 30 mg of TAM in 2 mL of corn oil (Sigma-Aldrich). Adult K8^flox/flox^ and K8^flox/flox^;Villin-CreER^t2^ mice were injected with 100 µL of TAM solution intraperitoneally once per day for three consecutive days, resulting in a daily dose of 1.5 mg TAM per mouse. Disease activity was monitored by observations of weight, stool consistency, and rectal bleeding in TAM-injected K8^flox/flox^;Villin-CreER^t2^, and K8^flox/flox^ mice as previously described (12). Stool consistency was scored as 1 = normal; 2 = formed but soft; 3 = slightly loose; 4 =liquid or unable to excrete. Samples were collected from two sets of mice (Fig. 1*A*); one set with seven mice of each genotype from which stool samples were collected daily from the start of the experiment (referred to as *set 1*), and another set with 3 mice/day/genotype that were euthanized daily from the start of the experiment (*set 2*) for tissue sample collection.

All mice were euthanized by CO_2_ asphyxiation, followed by intracardiac puncture if serum was collected. The colon was excised and the length was measured. Stool samples were snap-frozen and stored at −80°C for RNA analysis. Tissue samples were either snap-frozen to be stored in liquid nitrogen or fixed in 4% paraformaldehyde (PFA). Proximal colon (PC) and distal colon (DC) segments were collected separately for histology but pooled for RNA analysis. Crudely isolated colonic epithelium, as previously described (23), was collected for protein analysis.

### Histological Evaluation

Paraformaldehyde (PFA, 4%) fixed samples were embedded in paraffin, cut into 4-µm-thick sections, and stained with hematoxylin and eosin (HE) for histological analysis. Histological methods were performed by the Histology core facility of the Institute of Biomedicine, University of Turku, Finland. Stained samples were scanned using a Pannoramic 1000 Slide scanner (3DHISTECH, Budapest, Hungary) and analyzed with CaseViewer 2.4 (3DHISTECH) and QuPath 0.4.0 (24). The length of ten full crypts in PC and DC areas was measured for each mouse to calculate the mean crypt length. Crypt loss, defined here as luminal colon area with mucous erosion and disrupted crypt structures, was measured along the mucosae muscularis and was compared with its total perimeter (7). Crypt loss was determined from the average of independently measured results by two people, blind for the genotype.

### SDS-PAGE and Western Blot

Snap-frozen colon samples were homogenized on ice in 0.187 M Tris-HCl, pH 6.8, 3% SDS, 5 mM EDTA, 1× complete protease inhibitor cocktail (Roche, Basel, Switzerland) and 1 mM phenylmethylsulfonyl fluoride (Sigma-Aldrich, St. Louis, MO). The total protein concentration of samples was determined using a Pierce bicinchoninic acid (BCA) protein assay kit (Thermo Fisher Scientific, Waltham, MA). The samples were diluted to 5 µg protein/10 µL with 3× Laemmli sample buffer (30% glycerol, 3% SDS, 0.1875 M Tris·HCl, pH 6.8, 0.015% bromophenol blue, and 3% β-mercaptoethanol). Proteins were separated on 10% SDS-polyacrylamide gels in which *M*_W_ was estimated using iBright Prestained Protein Ladder (Thermo Fisher Scientific). Proteins were transferred to polyvinylidene fluoride (Thermo Fisher Scientific) membranes for Western blot analysis. Primary and secondary antibodies used are listed in Supplemental Table S1, and have been validated by the supplier, except for anti-K18 (25). Bands were quantified using ImageJ software (National Institutes of Health, Bethesda, MD) (26) and normalized to Hsc70. Uncut Western blot membranes are available at etsin.fairdata.fi repository (https://doi.org/10.23729/631ff119-814f-4521-a74a-4b56d2105fb5).

### Gene Expression Analysis

Colon tissue samples were homogenized and total RNA was extracted using a NucleoSpin RNA kit (Macherrey-Nagel, Düren, Germany), and total RNA from stool samples was isolated using a NucleoSpin RNA Stool kit (Macherrey-Nagel), according to the manufacturer’s protocols. Extracted RNA samples were quantitated using Nanodrop 2000 Spectrophotometer (Thermo Scientific) and reverse transcribed into cDNA using a cDNA synthesis kit (Promega, Madison, WI). Quantitative PCR on genes of interest was performed using QuantStudio 3 real-time PCR system (Applied Biosystems, Waltham, MA) with SensiFAST SYBR Hi-ROX Kit (Meridian Bioscience, Cincinnati, OH). Primers used are listed in Supplemental Table S2. Gene expression was normalized to *Actb* and quantified using the ΔΔCt method. To assess exfoliate quantity in stool, concentrations of selected mRNA in stool (*Actb*, *Krt19*, and *Vim*) were determined after normalizing Ct-values by stool pellet weight and total isolated RNA concentration of each sample [10^−Ct^/(*C*_RNAtot_ × *m*_stool_)].

### Fecal Calprotectin Analysis

Mouse stool samples weighing 27–45 mg were incubated in fecal calprotectin (FC) lysis buffer (0.1 M Tris-base, 0.75 M NaCl, 1 M urea, 0.01 M CaCl_2_, 0.01 M citric acid, and 5 g/L BSA, pH 8.0) at +4°C overnight, homogenized using the TissueRuptor II (Qiagen, Hilden, Germany) and centrifuged to isolate the lysate. Fecal calprotectin concentration was measured using the Mouse S100A8/S100A9 Heterodimer DuoSet ELISA kit (R&D Systems, Minneapolis, MN) according to the manufacturer’s instructions. Absorbance was measured using Wallac Victor2TM (PerkinElmer, Waltham, MA) and the results were normalized to stool pellet weight.

### Serum Cytokine Analysis

Concentrations of circulating IL-1β, IL-5, IL-6, IL-25, IL-22, interferon γ (IFNγ), TNFα, C-C motif chemokine 2 (CCL-2), and IL-18 were analyzed using a Procartaplex multiplex assay (Thermo Fisher Scientific) according to the manufacturer’s protocol. Serum samples were centrifuged (10 min, 9,600 *g*) to remove debris, and cytokine concentrations were measured using the Luminex 200 system (Luminex Corporation, Austin, TX). Results were quantified based on protein standards included in the assay kit.

### Statistical Analysis

**Figure 2.**
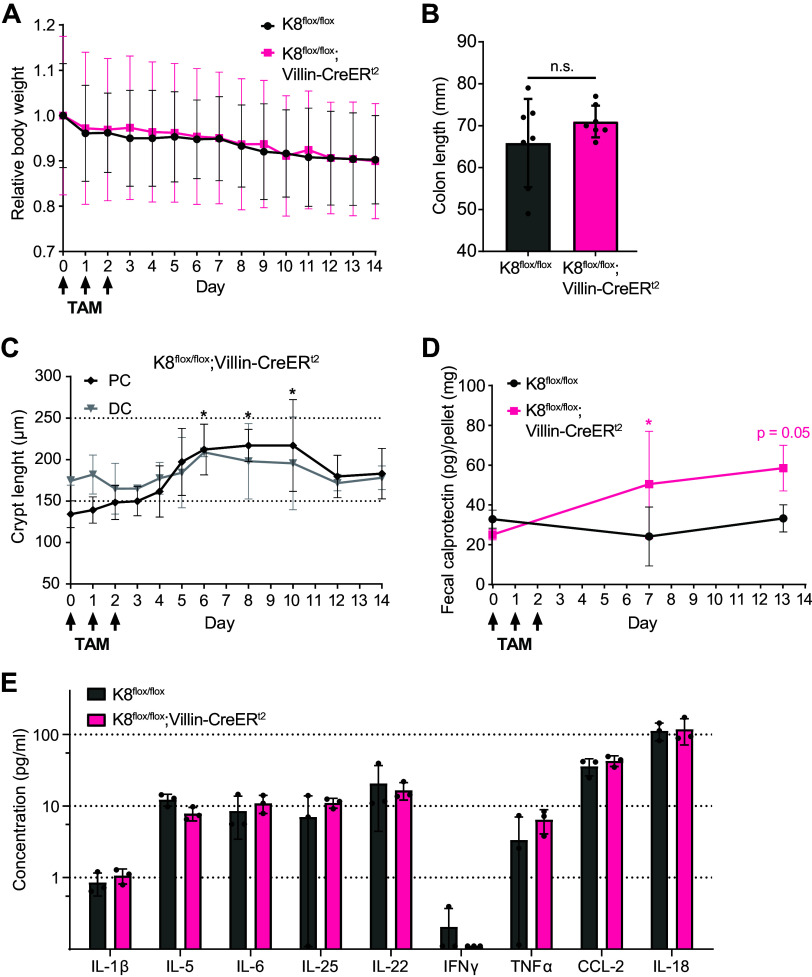
Targeted intestinal K8 loss induces crypt elongation and neutrophilic activity in mucosa without promoting systemic effects. *A*: K8^flox/flox^ and K8^flox/flox^;Villin-CreER^t2^ mice 0–6, 8, 10, 12, and 14 days following induction of K8 loss were analyzed for relative body weight change from *day 0*. Dots indicate mean change with whiskers representing SD. *B*: colon length was measured on *day 14* in K8^flox/flox^ and K8^flox/flox^;Villin-CreER^t2^ mice. Columns represent the mean length with whiskers representing SD and dots individual mice. *C*: hematoxylin and eosin (HE) staining from K8^flox/flox^ and K8^flox/flox^;Villin-CreER^t2^ mice 0–6, 8, 10, 12, and 14 days following induction of K8 loss were analyzed for proximal colon (PC) and distal colon (DC) crypt length. Dots indicate mean length with whiskers representing SD. *D*: stool lysates from K8^flox/flox^ and K8^flox/flox^;Villin-CreER^t2^ mice 0, 7, and 13 days following induction of K8 loss were analyzed for fecal calprotectin. Dots indicate mean fecal calprotectin quantity with whiskers representing SD. *E*: concentration of circulating serum cytokines IL-1β, IL-5, IL-6, IL-25, IL-22, IFNγ, TNFα, C-C motif chemokine 2 (CCL-2), and IL-18 were measured on *day 14* in K8^flox/flox^ and K8^flox/flox^;Villin-CreER^t2^ mice using Procartaplex multiplex assay. Results represent the mean concentration with whiskers representing SD and dots individual mice. Data in *A*, *B*, *D*, and *E* are from mouse *set 2* (*n* = 7, except *D*, *n* = 5; *E*, *n* = 3) with the same mice/time point, while data in *C* is from mouse *set 1* (*n* = 3). Statistical significance comparing all the other days to *day 0* was determined using two-way ANOVA Bonferroni’s post hoc test for *A*, *C,* and *D*, and statistical significance between groups determined using Student’s *t* test for *B* and *E*. Statistically significant differences are shown as *P < 0.05. TAM, tamoxifen.

**Figure 3.**
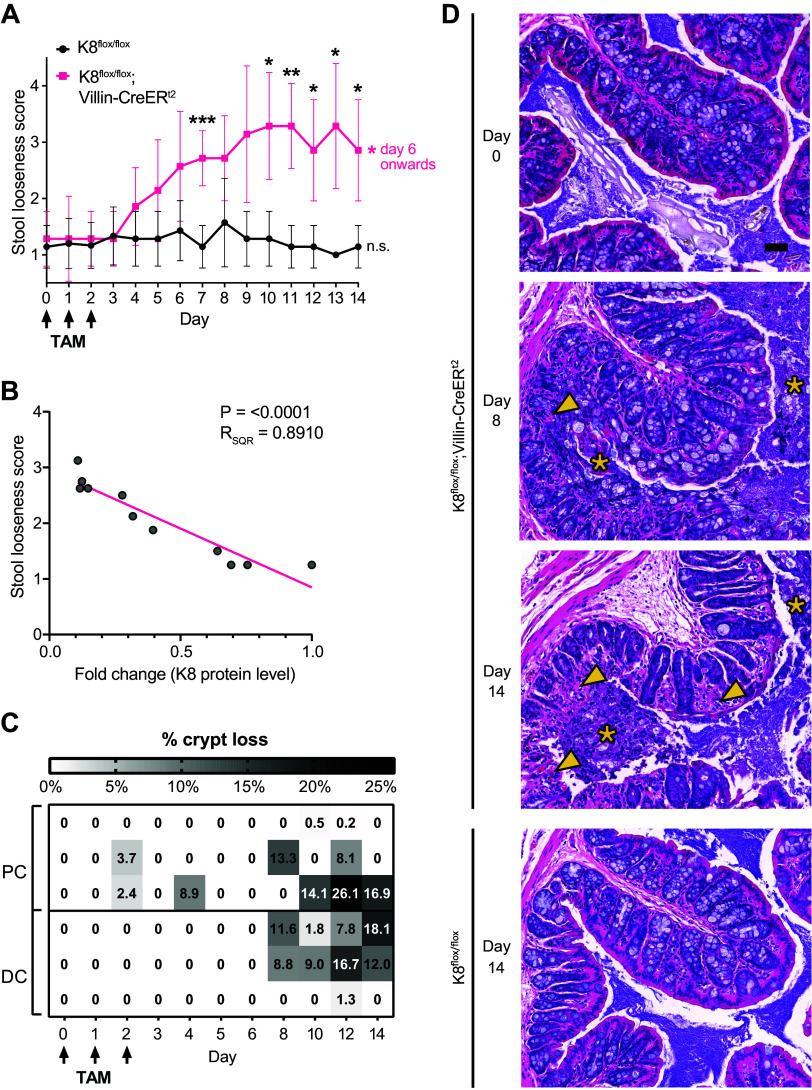
Intestinal-specific K8 knockout leads to diarrhea and loss of colonic crypts. *A*: K8^flox/flox^ and K8^flox/flox^;Villin-Cre-ER^t2^ mice 0–6, 8, 10, 12, and 14 days following induction of K8 loss with tamoxifen (TAM) were analyzed for stool looseness. Dots indicate mean value with whiskers representing SD. *B*: fold change of K8^flox/flox^;Villin-Cre-ER^t2^ K8 protein levels in stool correlate with stool looseness scores over the 14-day timeline following TAM administration. Dots indicate mean daily values. *C*: crypt loss in proximal (PC) and distal colon (DC) were analyzed on hematoxylin and eosin (HE) stained samples from K8^flox/flox^ and K8^flox/flox^;Villin-Cre-ER^t2^ mice 0–6, 8, 10, 12, and 14 days following induction of K8 loss. *D*: representative HE-stained samples of PC. Arrows indicate crypt loss and yellow stars indicate eroded epithelium in the lumen. Scale bar = 50 μm. Data in *A* and *B* are from mouse *set 1* (*n* = 7) with the same mice/time point, whereas data in *C* and *D* are from mouse *set 2* (*n* = 3). Statistical significance comparing all the other days to *day 0* (pink star) determined using two-way ANOVA Bonferroni’s post hoc test for *A* and *C*. Statistical significance comparing K8^flox/flox^ mice and K8^flox/flox^;Villin-CreER^t2^ mice (black stars) was determined using Mixed-effects analysis Bonferroni’s post hoc test for *A* and statistically significant differences are shown as *P < 0.05, **P < 0.01, and ***P < 0.001. Statistical significance of correlation was determined using simple linear regression (pink line) analysis for *B*.

**Figure 4.**
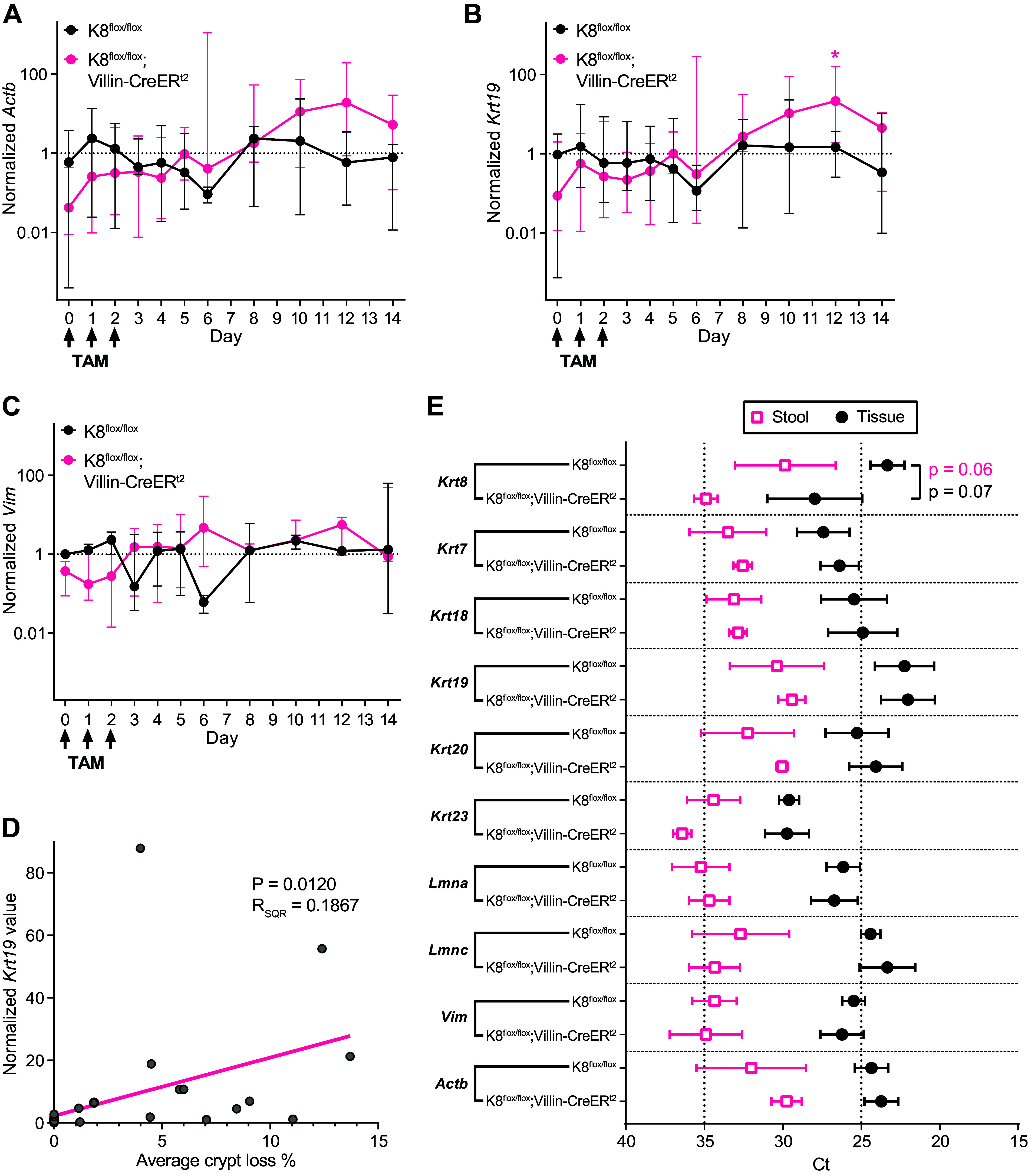
Colon epithelial cell gene expression and erosion can be noninvasively studied by stool sampling. Stool samples collected from K8^flox/flox^ and K8^flox/flox^;Villin-CreER^t2^ mice 0–6, 8, 10, 12, and 14 days following induction of K8 loss were analyzed for *Actb* (*A*), *Krt19* (*B*), and *Vim* (*C*) Ct values normalized to stool pellet weight and total RNA concentration. Dots indicate normalized median values with whiskers representing min/max. *D:* crypt loss percentage of K8^flox/flox^;Villin-Cre-ER^t2^ mice (Fig. 3*C*) correlates with normalized *Krt19* stool levels over the 14-day timeline following tamoxifen (TAM) administration. Dots indicate mean daily values. *E:* stool lysates and crudely isolated colon epithelial cells collected 14 days following induction of colon-specific K8 loss were analyzed for *Krt8, Krt7, Krt18, Krt19, Krt20, Krt23, Lmna, Lmnc, Vim,* and *Actb* Ct-values. Dots and boxes indicate mean Ct-value and whiskers represent SD. *n* = 7, except in *E*, where *n* = 3. Statistical significance comparing all the other days to *day 0* was determined using two-way ANOVA Bonferroni’s post hoc test for *A*–*C*, the statistical significance of the correlation was determined using simple linear regression (pink line) analysis for *D* and statistically significant differences between groups determined using Student’s *t* test for *E*. Statistically significant differences are shown as *P < 0.05.

GraphPad Prism V9.0 (GraphPad Software Inc., San Diego, CA) was used for statistical analyses. Statistical significance between two groups was determined using an unpaired Student’s *t* test. Statistical differences between multiple groups within a time course were determined using the analysis of variance followed by the Bonferroni post hoc test. For series with missing data points, mixed effects analysis was performed instead of an analysis of variance. Differences with a *P* value <0.05 were considered significant and significant differences between groups were presented as *P < 0.05, **P < 0.01, and ***P < 0.001. GraphPad Prism and Adobe Illustrator 2021 (Adobe, Inc., San Jose, CA) were used to generate Figs. 1–4; graphical abstract was created with BioRender.com (Science Suite Inc. Toronto, Canada).

## RESULTS

### Induction of Intestine-Specific K8 Deletion Leads to a Rapid Loss of Keratins, Detectable in Both Tissue and Stool Samples

Within one day following the first TAM injection to K8^flox/flox^;Villin-CreER^t2^ mice, a 60–70% loss of K8 mRNA was observed in both colonic epithelium and stool samples (Fig. 1*B*, Supplemental Fig. S1). Colon K8 protein levels were significantly, although modestly, downregulated on *day 1*; however, the decline was slower over the first 7 days compared with mRNA levels (Fig. 1*B*, Supplemental Fig. S1), as expected, reaching its lowest levels on *day 8* (∼90% loss of K8). K18 and K19 protein levels were significantly downregulated from *day 4* onward, without any changes in K18 and K19 mRNA levels (Fig. 1, *C* and *D*), as expected. K8 loss had no significant effect on K7 expression (Fig. 1*E*).

### Epithelial K8 Deletion Results in Crypt Elongation within 6–10 Days, Followed by a Modest, Local Inflammatory Response

Intestinal epithelial-specific K8 loss in K8^flox/flox^;Villin-CreER^t2^ did not affect body weight within 14 days following TAM administration (Fig. 2*A*). Within the two weeks studied, colon lengths were similar to control K8^flox/flox^ mice (Fig. 2*B*), and no rectal prolapses, occurring in germline K8 deletion (27) and adult K8^flox/flox^;Villin-Cre-1000 mice after several months (7) were observed. Crypt length, however, was steadily increasing over time in the PC, but not in DC, reaching statistical significance with approximately a 65% average increase in length within 6 days following the first TAM administration (Fig. 2*C*). The crypt length increase was preceded already from *day 2* by a significant decrease of the IL-22 receptor antagonist, IL-22BP (Supplemental Fig. S2), which is associated with balancing colonic epithelial proliferation and regeneration (28, 29). The increase in crypt length was also accompanied by modest neutrophilic activity in the colon as measured by an increase in fecal calprotectin (Fig. 2*D*). The neutrophil activity remained local, with no observed significant changes in circulating cytokine concentrations on *day 14* (Fig. 2*E*).

### Keratin Depletion Leads to Early Diarrhea and Later to Focal Crypt Loss

K8^flox/flox^;Villin-CreER^t2^ mice started developing diarrhea between *days 4* and *6* following K8 downregulation (Fig. 3*A*), and stool looseness scores strongly correlated with K8 protein downregulation (Fig. 3*B*). The first diarrheic mice were found on *day 4* when K8 levels were reduced by 50% compared with controls and a significant difference was observed after *day 6* when K8 levels were less than 30%. Keratin protein downregulation was accompanied by a decline of the ion channel transporter *Slc26a3* (DRA). However, its decrease failed to reach significance due to substantial variation (Supplemental Fig. S3). On *days 8* to *14* when K8 protein levels reached their lowest concentrations, areas with patchy epithelial loss were found in all parts of the colon, covering ∼6% of the epithelium. However, individual variation was high (Fig. 3, *C* and *D*). Crypt loss was accompanied by increased observations of exfoliated colonocytes inside the lumen (Fig. 3*D*).

### Stool qPCR Analysis Can Be Utilized to Follow Epithelial Changes Noninvasively in Sequential Intestinal Disease Models

qPCR-derived Ct-values reflecting the mRNA concentrations were normalized to pellet weight and total RNA concentration. *Actb* and epithelial *Krt19* mRNA content in fecal samples increased throughout the time course (Fig. 4, *A* and *B*), despite major individual variance likely due to patchy crypt damages (Fig. 3*C*). Meanwhile, the concentration of nonepithelial, type III IF vimentin (*Vim*) remained unchanged (Fig. 4*C*). A linear correlation between the normalized concentrations of fecal *Krt19* and the percentage of observed colonic crypt loss was identified, indicating elevated epithelial exfoliation around *day 12* (Fig. 4*D*).

Overall, stool samples’ Ct-values of IF genes were found to be comparable with colon tissue samples collected from the same mice (Fig. 4*E*). However, the Ct-values were systematically lower in tissue samples than in stool, indicating higher colon tissue mRNA concentrations as expected. TAM-induced *Krt8* downregulation in colon epithelium was similarly detectable in both tissue and stool samples (Fig. 4*E*). Ct-values for *Krt8* and its main binding partner in the intestine, *Krt19*, were the lowest, demonstrating the highest mRNA concentrations, closely followed by *Krt18* and *Krt20*, with *Krt7* and *Krt23* having the lowest mRNA concentrations out of the colonic keratins. *Vim* expression was low in both tissue and stool samples. Moreover, the difference in *Vim* Ct-values between tissue and stool samples was the highest among all measured IFs (Fig. 4*E*), indicating that low numbers of *Vim* expressing innate immune and mesenchymal cells are shed in stool compared with epithelial cells.

## DISCUSSION

Although previous studies from germline K8 knockout and tissue-specific K8-deficient mice have identified many colitis-related phenotypes induced by K8-deletion, the earliest phenotypes, and thus, evidence for the K8 primary functions, are elusive (4). We recently showed that K8-deletion in adult K8^flox/flox^;Villin-CreER^t2^ mice already at 25 days had complete loss of colonocyte K8 and developed comparable damage of the colonic epithelium and crypt length increases as in germline and tissue-specific mice (7). To identify major primary, early, and autonomous functions of colonic epithelial K8, we here performed daily phenotyping during 2 wk following the sequential downregulation of K8 in adult mouse colonic epithelial cells using the K8^flox/flox^;Villin-CreER^t2^ mice. We identified that diarrhea and increased crypt length preceded major epithelial erosion (Summarized in Table 1).

**Table 1.** Summary of TAM-induced significant changes in colon epithelia of K8^flox/flox^;Villin-CreER^t2^ mice during the timeline of 14 days, as demonstrated in colon and stool samples

Observed Changes in K8^flox/flox^;Villin-CreER^t2^ Mice Colon Epithelia after Tamoxifen Injection	*Day*											
	*0* ^a^	*1* ^a^	*2* ^a^	*3*	*4*	*5*	*6*	*7/8*	*9/10*	*11/12*	*13/14*	*25* ^b^
Colon tissue												
Tissue K8 mRNA↓		***	***	***	***	***	***	***	***	***	***	***
Tissue K8 protein↓		**	***	*	***	***	***	***	***	***	***	***
Tissue K18 protein↓					***	***	***	***	***	***	***	**
Tissue K19 protein↓					**	***	***	***	***	***	***	**
Tissue IL-22BP protein↓			***	***	***	***	***	***	***	***	***	***
Crypt length↑							*	*	*			***
Crypt loss↑												**
Stool												
Stool K8 mRNA↓		***	***	***	***	***	***	***	***	***	***	n.d.
Stool looseness↑							*	*/*	***/***	***/**	***/**	n.d.
Stool calprotectin↑								*			0.05	n.d.
Epithelial cell exfoliation↑										*		n.d.

N.d., not determined; TAM, tamoxifen.

^a^ Tamoxifen injection administered; ^b^Data from Stenvall et al. (7), tamoxifen-treated K8^flox/flox^;Villin-CreER^t2^ mice compared with untreated K8^flox/flox^;Villin-CreER^t2^ mice. Statistical significance comparing all the other days to *day 0* is shown as *P < 0.05, **P < 0.01, and ***P < 0.001 where the arrows indicate the overall direction of a change: ↑, increasing; ↓ decreasing.

*Krt8* deletion in adult K8^flox/flox^;Villin-CreER^t2^ mice stimulated colonocyte proliferation and led to crypt erosion in the colon within 6–12 days following induction, focally exposing the mesenchyme. Colonocyte hyperproliferation in mice with germline K8 loss has been associated with increased IL-22 signaling through a near-complete loss of IL-22BP expression and activated STAT3 signaling (7, 28). Here, we show that the sequential downregulation of K8 leads to a very early and persistent decrease in IL-22BP, suggesting a role for K8 in colonocyte proliferation, although the exact mechanisms warrant further studies. The percentage of cryptless areas on *days 10* to *14* was analogous to that found in mice with germline K8 deletion (7), indicating that the keratin-deficient colon phenotype stabilizes already within 14 days after *Krt8* deletion. Therefore, approximately three to four cycles of crypt renewal are needed to achieve a stable phenotype, as the mean lifespan of mouse colonocytes past the stem cell phase is around 3 days (30). Although K8 mRNA levels were immediately suppressed after the onset of K8-loss by TAM administration, downregulation of intestinal keratin protein expression was slower, in concordance with the expected colonocyte renewal rate. The half-life of epithelial cell keratins has not been widely studied; however, the K8 and K18 half-life in hepatocytes clearly exceed 24 h (31), which is consistent with our finding, indicating that the same keratin protein units may remain in differentiated colonocytes during their whole lifecycle. Once the K8 protein level dropped more than 30%, mice started to exhibit loose stool, along with elongation of colonic crypts. During the latter half of the 14-day time course, cells began to sporadically detach, leading to luminal areas lacking conventional crypt structures, leading to increased mesenchyme exposure. This further resulted in increased levels of FC, reflecting mucosal neutrophil activity (Table 1).

Along with histological staining demonstrating an increased presence of luminal exfoliated cells toward the latter part of the time course, the concentration of epithelial cell-derived mRNA was elevated in the feces on *day 10* and *12*, indicating increased epithelial cell content. The average fecal *Krt19* concentration was over 20-fold higher on *day 12* compared with *day 0*, and fecal *Actb* concentration profile was similar to the *Krt19* profile. On the contrary, the fecal *Vim* profile was unchanged during the 14-day time course, suggesting that primarily only colonocytes detach and exfoliate (32). In addition, the IF gene expression profiles in crypts were comparable with fecal profiles on corresponding time points, indicating that colonic epithelial events such as crypt loss can be studied in exfoliated cells and cell components in feces.

Focal cryptless areas found after K8 deletion are likely a consequence of increased intestinal cell detachment. This could be attributed to a weakening of the cytoskeletal network and their connections with desmosomal junctional complexes, which facilitate mechanical colonocyte attachments in which keratins are among the key components (10, 11, 13, 14). Similarly, intestinal epithelial plectin or α6-integrin ablations have been associated with reduced colonocyte capacity to resist mechanical stress and a colonic phenotype with notable similarities to K8^flox/flox^;Villin-Cre mice. We could not identify why the crypt length increase was more pronounced in the proximal parts of the colon compared with the distal parts; however, the proximal colon was more affected during keratin deficiency-induced inflammation (19). In addition, the distribution of innate immune cells varies between colon regions (33), which may explain the difference in rapid cellular responses. Leukocyte activity could also induce erosion, but the fecal calprotectin concentrations increased less than twofold after K8-deletion, indicating a minor recruitment in neutrophil numbers in this relatively short and acute 2-wk study protocol. In acute colitis models with severe erosion and fibrosis, such as DSS mice and IL-11 transgenic mice, substantially higher calprotectin concentration is reported (34, 35). A limited role for leukocytes during the first 2 wk following K8 deletion in the K8^flox/flox^;Villin-CreER^t2^ model is supported by a lack of systemic immune responses, in line with what has previously been observed in the germline conditionally K8-depleted K8^flox/flox^;Villin-Cre-1000 mouse model (7).

Results in this study show that the *Krt8* deletion progress can be followed nearly in real time solely by studying stool samples, which can be useful to follow conditional gene silencing and for preclinical IBD models where the epithelium is partly damaged and exfoliated. The noninvasive methodology might be useful for colon cancer models, e.g., CDX2, villin, and APC expression-based models (36), which often are used in long-term experiments, with few options for noninvasive monitoring of early tumor development. Using noninvasive stool samples instead of daily euthanasia strongly promotes 3 R principles in the ethical use of experimental animals (37). The presence of exfoliated cells in stool has been known for a long time, and their amount and morphometry have been associated with some diseases, especially gut adenocarcinomas (38). The first indication that loose cells could be used to diagnose gut malignancies was published already in the 19th century (39, 40). Exfoliated cells in stool are sometimes studied in clinical research (41–43), but the utilization of this method in preclinical work has been limited, although methodologies were already introduced in the 1990s (44). Fecal murine RNA levels have been used to evaluate colonic cytokines in CRC (45) and investigate intestinal gene expression (46) or genotype in mice (47). Furthermore, a recent study showed that K8 protein is measurable in fecal samples of patients with necrotizing enterocolitis (48). To corroborate further development of these methodologies, we show here that fecal mouse mRNA analysis using a NucleoSpin RNA Stool kit (Macherrey-Nagel) is an effective, noninvasive method that can be used for frequent quantitative or semiquantitative monitoring of epithelial gene expression and colonic epithelial changes. In addition, fecal sampling allows for individual monitoring of single mice over time. Potentially, this warrants future clinical translation of this methodology for, e.g., screening of chronic gut diseases.

One of the earliest phenotypes following the reduction of K8 levels identified in this study is mild diarrhea. Colon water balance is mainly regulated by chloride and sodium ions, and a defective colonocyte ion exchange disrupting water uptake has been described in the whole body K8-knockout mice (49, 50). Especially, the downregulation of DRA, a central chloride transporter protein, was dramatically downregulated in these mice on protein and mRNA levels (50). Furthermore, mice with only one intact K8 allele (and intact epithelium) displayed partially disrupted ion exchange (43), and decreased and patchy DRA protein without effects on DRA mRNA (50). Further supporting our data that K8 has a primary role for colonocyte ion transport is that loose stool was observed in the whole body K8-knockout mice 2 days after birth (49). Although we did not find a statically significant decrease in DRA mRNA levels in the present study, the average levels were reduced and further molecular and mechanistic roles of K8 for ion transporter functions are warranted.

K8-deficient mice have been suggested to model IBD (16). However, although the phenotype exhibited by K8^flox/flox^; Villin-CreER^t2^ mice in this study shared certain characteristics with early IBD, this resemblance is limited by a lack of a pronounced immune response. Comparatively, in IBD, immune cell infiltration and distorted crypt architecture are pronounced even before the appearance of symptoms (22). We recently demonstrated that K7 is neo-expressed in patients with IBD (51), but K7 was not markedly changed after K8 deletion in the present preclinical study. A study including over 900 patients with IBD found that common *KRT8* and *KRT19* variants are neither overtransmitted nor associated with IBD (52). Although modest variation in keratin colon expression has been reported between different subtypes and activity phases in IBD (53), and models of colonic stress (23), the role of IFs in IBD initiation and activity still requires further study. Nevertheless, parallels can be drawn between the phenotype of K8^flox/flox^;Villin-CreER^t2^ mice, and various diarrheic diseases other than IBD, such as microscopic colitis (54), leaky gut, viral and bacterial diarrhea. Similarities include constant, but not life threatening, diarrhea, modest neutrophil activity, and minor focal changes in crypt histomorphometry (55–58). Not much is known about the possible keratin changes in diarrhea, microscopic colitis, irritable bowel syndrome, or colon functionality in general. Colon keratin research has focused mainly on chronic, potentially lethal diseases, most importantly colon cancer (4). Thus, the suitability of K8^flox/flox^;Villin-CreER^t2^ mice to model diarrheic diseases and the relationship between keratin expression and colon health warrants further research.

In conclusion, we show here that conditional ablation of K8 in intestinal epithelial cells leads to immediate changes in colonocytes, establishing a phenotype commenced by diarrhea and increased proliferation signaling leading to increased crypt length, followed by epithelial erosion and ultimately resulting in exposure of the mesenchyme and increased intestinal neutrophilic activity. In addition, we present a noninvasive method for daily monitoring of crypt loss in preclinical in vivo models.

## DATA AVAILABILITY

The raw numeric data, uncut membranes and datasets generated during this study are available in the etsin.fairdata.fi repository at https://doi.org/10.23729/631ff119-814f-4521-a74a-4b56d2105fb5.

## SUPPLEMENTAL DATA

10.23729/3eec5520-7aa4-4d3d-a00b-9e39bddc942f Supplemental Figs. S1–S3 and Tables S1 and S2: https://doi.org/10.23729/3eec5520-7aa4-4d3d-a00b-9e39bddc942f.

## GRANTS

This study was supported by Academy of Finland project Grants 315139, 332582 including InFLAMES Flagship Programme, 337531 357911 (to D.M.T.); Åbo Akademi University Center or Excellence in Mechanostasis, and Solutions for Health (to D.M.T.); Medicinska understödsföreningen Liv och Hälsa foundation (to D.M.T.); Suomen Kulttuurirahasto, Varsinais-Suomi Regional Fund, 85222249 (to M.T.); Swedish Cultural Foundation (to M.A.I. and C.-G.A.S.), Victoriastiftelsen (to M.A.I. and C.-G.A.S.); Agneta and Carl-Erik Olin foundation (to C.-G.A.S.); K. Albin Johansson foundation (to C.-G.A.S.); and National Heart, Lung, and Blood Institute Grant HL154998 (to K.M.R.).

## DISCLOSURES

No conflicts of interest, financial or otherwise, are declared by the authors.

## AUTHOR CONTRIBUTIONS

M.A.I., L.P., C.-G.A.S., K.M.R., and D.M.T. conceived and designed research; M.A.I., L.P., C.-G.A.S., M.T., K.K., and D.M.T. performed experiments; M.A.I., L.P., C.-G.A.S., M.T., and K.K. analyzed data; M.A.I. and L.P. interpreted results of experiments; M.A.I. and C.-G.A.S. prepared figures; M.A.I. and L.P. drafted manuscript; M.A.I., L.P., C.-G.A.S., M.T., K.K., K.M.R., and D.M.T. edited and revised manuscript; M.A.I., L.P., C.-G.A.S., M.T., K.K., K.M.R., and D.M.T. approved final version of manuscript.
